# Effect of Ultralight Filler on the Properties of Hydrated Lime Injection Grout for the Consolidation of Detached Historic Decorative Plasters

**DOI:** 10.3390/ma13153360

**Published:** 2020-07-29

**Authors:** Andreja Padovnik, Violeta Bokan-Bosiljkov

**Affiliations:** Faculty of Civil and Geodetic Engineering, University of Ljubljana, Jamova 2, SI-1000 Ljubljana, Slovenia; violeta.bokan-bosiljkov@fgg.uni-lj.si

**Keywords:** detached decorative plasters, injection grout, glass microspheres, reduced density, stability, strength, durability

## Abstract

Injection-grout density is an important parameter when its additional weight leads to consolidated decorative plasters becoming damaged. This is especially evident in larger detached areas. In this study, thin-walled soda–lime–borosilicate glass microspheres were used as a density-reducing constituent in hydrated lime grout mixtures. The normal density grout composition—one volume part hydrated lime and three volume parts inert limestone filler with 0.5% of the polycarboxylate ether-based superplasticiser—was modified with partial substitution of the limestone filler with lightweight glass microspheres. The following volumetric proportions between limestone filler and glass microspheres were used: 100%:0%, 67%:33%, 50%:50%, 33%:67%, and 0%:100%. With the increase of the glass microspheres’ volume, the density of the grout is gradually reduced. Furthermore, there is a decrease in the stability and injectability of fresh grout. In its hardened state, the grout’s strength again reduces gradually, but there is no significant change in the grout’s water absorption and water-vapour resistance. The resistance of the grout to freezing–thawing and heating–cooling cycles using distilled water or salt solution is highly improved when the microspheres are present.

## 1. Introduction

Consolidation of detached plasters with historic value is an irreversible conservation treatment for re-establishing adhesion between delaminated decorative layers. Injection grouts, used for stabilisation of decorative plasters, have to be compatible with original historical materials. Furthermore, they should provide adequate flowability, injectability, and stability in a fresh state. Requirements regarding the grouts’ hardened state are very often given in relation to the properties of detached historical plaster; they should exhibit similar dry porosity, water-vapour permeability, capillary water absorption, and mechanical strength. An example of such requirements summarised by Padovnik et al. [1] is given in Table 1.

Additionally, specific properties, such as a low density of the grout, its increased durability, etc., are sometimes required. For larger detachments, a high quantity of grout is used to re-establish adhesion between the delaminated layers; low density of the grout is a key property that must be prescribed to prevent the formation of new damage and/or falling of the plaster from wall or ceiling, during or after consolidation by grout injection. Said density can be considerably reduced by the incorporation of lightweight filler to the grout mixture. The types of fillers used for such purpose are mineral materials with high porosity and water absorption, such as pumice, perlite and expanded glass or clay aggregate [2,3], and glass microspheres [4,5]. Very often, however, a composite filler—a mixture of pumice and glass microspheres—is used in conservation practice [6,7,8].

The glass microspheres—also known as glass bubbles—are nonabsorbent and have a significant advantage over porous fillers, since they can preserve extremely low wet and dry densities. Their spherical morphology, regular surface texture and extremely small particle size can help achieve and maintain suitable viscosity and stability, as well as improved injectability of the grout [4,7]. Zajadacz and Simon [4] studied the grout composition with Scotchlite Bubbles and Silcosil (fine-ground silica). They attributed the improved injectability to the small particle size of the glass microspheres and silica filler. The glass microspheres were well dispersed inside the grout mixture and tended not to segregate from other constituents, resulting in improved stability. The viscosity of this grout, however, was higher than usual. Rickerby et al. [7] used a high proportion of glass microspheres in a grout based on mud and pumice. They found that the spherical morphology of the microspheres contributed to poor packing ability and reduced internal cohesion of the grout mixture. Furthermore, Rousset et al. [9] reported that injection grout, prepared using a hydrated lime binder and glass microspheres as filler, possessed lower shrinkage and good adhesion. In Pasian et al. [8], microstructural analyses of the grout, based on slaked lime, pumice powder, and glass microspheres, showed the presence of hydraulic components on the microspheres’ surface; a result of the pozzolanic reaction. Additionally, it was evident from the backscattered electron images of the cross sections and broken sections that some glass microspheres in the studied grouts were broken.

When using the glass microspheres as a weight-reducing filler in a hydrated lime grout composition, the viscosity of the paste must be high enough to prevent the segregation of solid particles. To provide both an increased viscosity and adequate injectability of the hydrated lime grout, superplasticiser (SP; polymeric admixture) can be incorporated into the grout mixture [13,14]. Subsequently, much lower water content is needed for adequate workability and injectability. Among three SP groups frequently used in the lime-based grouts—poly-naphthalene sulfonates (PNS), lignosulfonates (LS) and polycarboxylate ethers (PCE)—the PCE products seem to be the most efficient solution [1,13].

The objective of this study was to develop a low-density hydrated lime injection grout, possessing adequate properties in both its fresh and hardened state in order to be used for the consolidation of detached lime plasters with historical value. Additionally, the grout was to be resistant to freezing–thawing and heating–cooling cycles, if possible, also in the presence of salts. The reduced density of the grout was obtained by using glass microspheres in the composite filler, which also contained fine-ground limestone particles. The required viscosity of the lime paste—to prevent segregation of filler particles and provide adequate injectability of the grout—was achieved using a combination of reduced water content and PCE superplasticiser. A parametric study was carried out as a means of determining the optimal composition of the lightweight grout, which would, in turn, ensure its improved durability.

## 2. Materials and Methods 

Commercially available, dry hydrated lime of the class CL 90-S (standard EN 459-1 [15]) was used as a binder, with its density being 2.22 g/cm^3^. Finely ground limestone (hereafter limestone filler or LS) composed of 95.3% calcite and 4.7% dolomite was selected as the main filler. The limestone filler particles had a density of 2.76 g/cm^3^; their maximum size was 100 µm, with 10%, 20%, 50%, and 90% of particles smaller or equal to 3 µm, 9 µm, 15 µm, and 40 µm, respectively. The water absorption of LS was 0.5%. The chemical compositions of the binder and limestone filler, determined by the X-ray fluorescence analysis (Bruker S8 TIGER, Anhovo, Slovenia) according to the EN 196-2:2013 standard [16], are given in Table 2. 

Thin-walled soda–lime–borosilicate glass microspheres (3M Glass Bubbles K1) were used as a density-reducing constituent of the grout, with their typical density being 0.125 g/cm^3^ and respective minimum and maximum densities being 0.10 g/cm^3^ and 0.14 g/cm^3^. The maximum size of the microspheres was 120 µm, with 10%, 50%, and 90% of particles smaller or equal to 30 µm, 65 µm, and 115 µm, respectively.

In order to obtain the adequate viscosity and injectability of the grout in a fresh state, a polycarboxylate ether-based superplasticiser (PCE-SP) with a relative density of 1.05 g/cm^3^ ± 0.02 g/cm^3^ and a pH value of 5.5 ± 1.0 was used as a highly efficient water-reducing agent. 

All materials were stored in a room at a controlled temperature of 20 °C ± 1 °C and relative humidity of 60 ± 5%. The grout mixtures were prepared using tap water at a temperature of 20 ± 1 °C. 

In previous research [1], the grout mixture based on 1 volume part hydrated lime and 3 volume parts limestone filler, with the addition of the PCE-SP chemical admixture, showed the best behaviour in the fresh and hardened states. This grout composition was selected as the normal density reference mixture to which properties of compositions with reduced densities were compared. Grouts with reduced densities were designed in such a way that the gradually increasing part of the limestone filler in the reference mixture was replaced with the same volume of glass microspheres. Consequently, five different grout compositions were obtained (Table 3), with the following volumetric proportions between limestone filler and glass microspheres: 100%:0%; 67%:33%, 50%:50%, 33%:67%, and 0%:100%. 

Two parameters of the reference grout mixture remained unchanged throughout different grout compositions: the hydrated lime content and the dosage of PCE-SP, calculated as a percentage of the total solid materials mass, i.e., the binder and the fillers, which was 0.5% [1]. The water content of the mixtures was adjusted to obtain adequate workability of each particular injection grout. The workability was evaluated by conservator via injection of the grout through a 10 mL syringe by applying minimum pressure, using the procedure described in [1].

### Mixture Preparation and Testing Methods

The grout mixtures were prepared with a simple handheld kitchen mixer, in order to simulate the preparation of injection grouts in the field. The mixer had a power of 300 W and five different mixing velocities. The metal whisk used was 8.5 cm long with a diameter of 4.6 cm. For compositions with glass microspheres, the microspheres were first mixed with 50% of the water content into a slurry. The binder and the limestone filler were then dry-mixed for 15 s at the low speed of 540 rpm. During the next 45 s (at 540 rpm), the microspheres slurry and 20% of the water content were added and mixing at low speed proceeded for a further 45 s. During the last 15 s of mixing at low speed, the PCE-SP and the remaining 30% of the water content were added to the mixture. After that, the mixing was stopped, and the sides of the mixer bowl were scraped; the mixer was then turned on again and mixing proceeded for 3 min at medium speed (1200 rpm).

For nonstructural grouts, several adaptations of commonly available standard test methods were needed. We followed the testing procedures proposed by Biçer-Şimşir and Rainer [17] or Padovnik et al. [1]. A brief description of the testing methods is given in the continuation. The tests were carried out in the laboratory with controlled temperature (20 ± 1 °C) and relative humidity of the air (60 ± 5% RH). First, the methods to evaluate fresh grouts’ properties are given, followed by the tests carried out on hardened grouts.

The wet density of each grout was determined according to an adapted EN 1015-6 [18] standard procedure. The volume of the mixture was reduced from 1000 mL to 100 mL, using a metal cylindrical vessel. The filling of the measuring vessel was carried out in the same manner as in the case of soft mortar [18]. The wet density was calculated as a quotient of measured mass of the grout and the 100 mL volume. 

The mini slump flow test [19] was used to determine the consistency of the grouts. A truncated cone-shaped mould (according to EN 459-2 [20]) placed at the centre of a smooth plate was filled with fresh grout. The average spread of the grout after lifting the mould was measured [1].

A bleeding test was carried out according to the adapted ASTM C940 [21] standard procedure. The volume of grout used in the test was reduced from 800 ± 10 mL to 80 ± 1 mL. Apart from this, the standard procedure was followed. A graduated cylinder of 100 mL was filled with 80 mL of the grout. Change in the accumulation rate of bleed water on the surface of the grout was observed over a period of time. The bleeding was calculated as a quotient between the volume of final bleed water and the initial volume of the grout. 

The water-retaining ability of fresh grout was evaluated by the standard procedure prEN 1015-8:1999 [22,23]. The fresh grout was subjected to suction provided by filter papers, resulting in a loss of water. The mass of water remaining in the grout was expressed as a percentage of the grout’s initial water content and reported as the water retentivity.

The injectability test was carried out according to the adapted standard procedure EN 1771:2004 [24]. Crushed lime mortar was used as a granular material, with a water absorption coefficient of 1.4 kg/(m^2^∙s^½^) after 10 min, and total and capillary porosities equal to 27% and 26%, respectively. The cumulative amount of the granular material passing through 1 mm, 2 mm, 3 mm, and 4 mm sieves was 5%, 12%, 42%, and 99%, respectively [1]. This test determined the ability of fresh grout to fill a capillary network of granular material in a dry or prewetted state.

The drying shrinkage test with mortar cups was used to determine the reduction in grout volume after drying. The procedure proposed in [17] was applied. The mortar for the mortar cups was prepared with 1 volume part lime putty and 2 volume parts limestone sand (0/1 mm). It had a water absorption coefficient of 1.8 kg/(m^2^∙s^½^) after 10 min, and the total and capillary porosities equal to 32% and 25%, respectively. Each cup had an outer diameter of 75 mm and a height of 30 mm. Dry and prewetted mortar cups were filled with grout mixtures; dimensional changes, as well as the crack-pattern development, were observed as the grout dried at 20 ± 1 °C T and 60 ± 5% RH.

**Figure 1:**
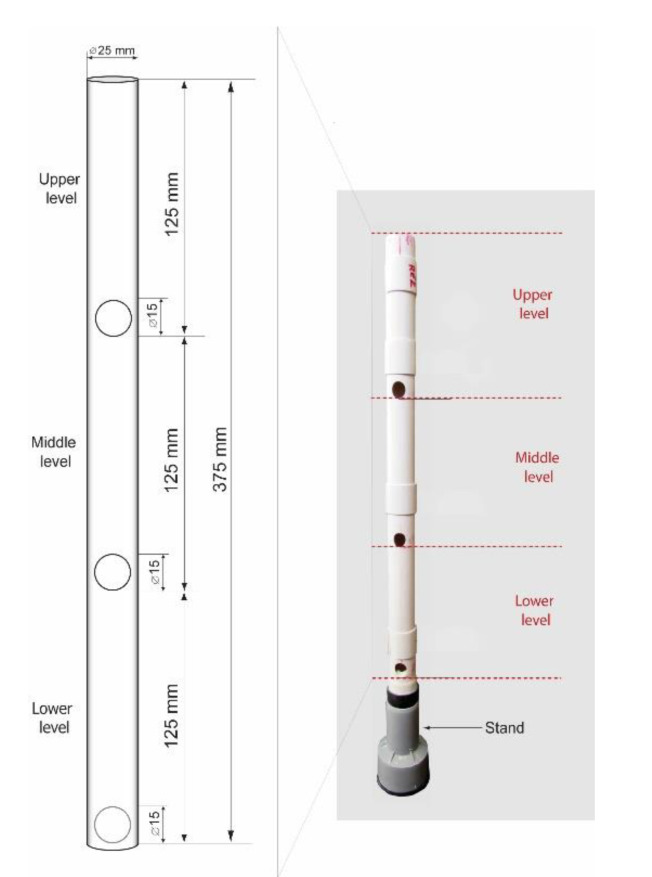
Cylindrical plastic tube dimensions.

The adapted settlement column segregation test [25] was used in order to assess the stability of fresh grout mixtures. A cylindrical plastic tube with an internal diameter of 22 mm and a height of 375 mm with three holes to facilitate the collection of a sample from the top, middle, and bottom levels (Figure 1) was filled with fresh grout and covered. After an hour, a sample from the top of the column was transferred into the first glass container. The same procedure was applied for the middle and bottom levels. For each of the three levels, the wet density of the collected sample was determined according to the adapted standard procedure EN 1015-6 [18], previously described. The stability of fresh grout is of utmost importance for the compositions where a combination of glass microspheres and superplasticiser is used, since such mixtures are particularly sensitive to segregation.

In order to determine the properties of the grouts in their hardened state, cylindrical moulds were used to cast the specimens (at least three samples for each property and age). They were left in the moulds for 48 h and subsequently cured under controlled laboratory conditions (relative humidity 60 ± 5% and temperature 20 ± 1 °C) until testing. The hardened properties were determined at the grouts’ age of 90 days, with compressive strength being measured again at 365 days. 

The dry density was determined according to EN 1015-10 [26], on 50/50 mm cylinders. 

The total and capillary porosities of the 50/50 mm cylinders were evaluated according to the Swiss standard SIA 262/1:2003, Appendix A [27]. Each specimen was subjected to different intensities of water saturation. Total and capillary porosities were calculated from the test results. 

Water absorption by capillarity was measured following RILEM test No. II.6 [28]. Dry 50/50 mm cylinders were placed 2 mm deep in water and weighed at the prescribed intervals. The weight change of the specimen was used to calculate the amount of water absorbed after a predetermined time; subsequently, water absorption coefficients after 10 min and 24 h were calculated.

Water-vapour resistance coefficient (μ) was determined according to standard EN ISO 12572:2001 [29], using the dry cup method. A cylindrical specimen, with a diameter of 100 mm and height of 20 mm, was put on top of a vessel (cup) containing a desiccant (calcium chloride, CaCl_2_), sealed to the vessel’s rim, and placed in a humidity-controlled chamber at 23 ± 0.5 °C and 60 ± 3% RH. The rate of water-vapour transmission through the specimen from the controlled atmosphere to the inside of the cup was determined by periodic weighing of the cup with the desiccant and the specimen.

The compressive test was carried out according to the EN 1015–11/A1 procedure [30], on 50/50 mm cylinders. 

The splitting tensile test was carried out according to the ASTM C496/C496M-11 procedure [31], on 50/50 mm cylinders. 

**Figure 2:**
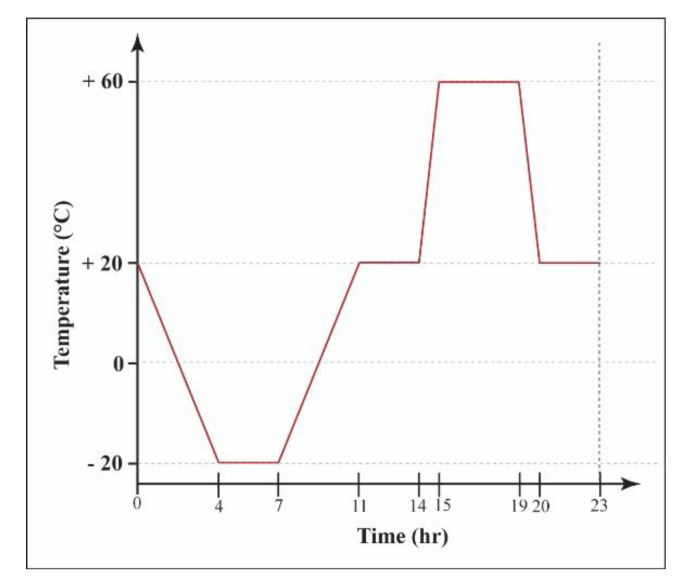
The protocol of the freezing–thawing and heating–cooling cycle used in the study.

The accelerated ageing was carried out on 50/50 mm cylinders, in order to assess the grouts’ durability. The specimens were subjected to fourteen freezing–thawing and heating–cooling cycles, following the protocol in Figure 2. Before each cycle, the specimens were subjected to capillary absorption of 3% NaCl solution or distilled water for 30 min, following the RILEM test No. II.6 [28], described earlier. Results of the capillarity water absorption test showed that the amount of water absorbed after 30 min was close to the value measured at 24 h. 

Finally, to evaluate the re-attachment ability of the grout, a pull-off test—according to the standard EN 1015-12 [32]—was performed on panel sandwich models [1]. The models were prepared to simulate a smaller (2 mm) and a larger (5 mm) detachment of fine plaster (1:3 lime putty: fine sand 0/1 mm lime mortar) from the rough plaster (1:3 lime putty: coarse sand 0/4 mm lime mortar). At the age of one year, the simulated detachments were filled by the grout using a syringe.

## 3. Results and Discussion 

### 3.1. Fresh State Properties 

Fresh grout properties for different limestone filler/glass microsphere ratios are presented in Table 4. 

The wet densities of fresh grouts confirm that the glass microspheres are an efficient constituent used to reduce the grout’s density. As expected, the lowest wet density was obtained for the grout GM_100_ (0.82 g/cm^3^), where only glass microspheres were used as filler material, and the highest value was obtained for the grout LS_100_ (1.73 g/cm^3^), where the limestone filler was the only mineral admixture. 

The wet densities of grouts with composed limestone–glass microspheres filler lie between these two limits, their average values being that of 1.51 g/cm^3^, 1.31 g/cm^3^, and 1.14 g/cm^3^ for the LS_67_-GM_33_, LS_50_-GM_50_, and LS_33_-GM_67_ grout, respectively. The replacement of the limestone filler of a relatively high density (2.75 g/cm^3^) with the same volume of glass microspheres of an extremely low typical density (0.125 g/cm^3^) is the main parameter that governs the grout’s weight reduction. The water content, which decreases with the increase in the glass microspheres’ volume (Table 3), is one additional parameter that influences the volume of prepared grout. Another is the packing density of solid particles in the suspension; grain size distribution of the glass microspheres is much coarser compared to the limestone filler. When considering the reduced weight of the grout, the two additional parameters—water content and packing density—need to be considered as well. Based on obtained results, it is possible to conclude that the reduction of the grout’s wet density with the incorporation of a relatively high volume of glass microspheres could be an effective method for reducing the weight of the grout, when the re-attachment of large plaster detachments needs to be carried out. 

The mini-slump-flow value of the fresh grout evaluates its flowability under the action of self-weight. It is a measure for fresh grout consistency, which is often related to the grout’s workability. In this study, the workability was evaluated via injection of the grout through a 10-mL syringe while applying minimum pressure [1], since the test method was set to reflect the conditions on the conservation site. The water content of the grout was reduced with the increasing volume of glass microspheres (Table 3), to obtain the same workability of the grouts. It is highly likely that with this, yield stress and viscosity of the lime paste (lime + water + PCE-SP) were increased as well. Comparing the consistency of the LS_67_-GM_33_ grout to that of the LS_100_ grout, a considerable increase in the slump-flow value could be observed. Slump-flow values of the LS_50_-GM_50_ and LS_33_-GM_67_ grouts were the same as that of the LS_100_ grout; these two compositions show the same workability as the LS_100_ grout and, consequently, also the same slump-flow value. Mini-slump-flow value is often related to the paste’s yield stress τ_0_; paste is a generic name for the mixture of binder, filler particles smaller than 0.1 mm and water, and can also contain a chemical admixture. The value of τ_0_ increases with an increase in paste density and decreases with the mini-slump-flow value increase. If we assume that only the paste’s own weight is controlling the phenomena, the equation proposed in [33] is the following: τ_0_ = C·ρ/SF^5^. In this equation, C represents a constant that includes gravity and volume of the paste, ρ is the paste’s density, and SF is the mini-slump-flow value. The equation can be used to explain the influence of the glass microspheres on the rheological properties of the grouts. It is clear that by replacing the fine limestone filler with coarser glass microspheres, the grout’s yield stress (τ_0_) decreases, despite the increase in yield stress and viscosity (µ) of the lime paste in the grout. For the constant SF value, the yield stress decrease is higher for grouts with a higher content of glass microspheres. The effect of glass microspheres on the rheological properties of the grout seems to be similar to that of air bubbles that are produced using an air-entraining agent (chemical admixtures) in cement paste. For the LS_50_-GM_50_ grout, the obtained standard deviation of the test results was relatively high. Visual observation of the grout spreads revealed segregation between solid particles during the test; heavier particles settled to the bottom and lighter particles (the glass microspheres) accumulated on the fresh mixture’s surface. A poor packing density of solid particles in the LS_50_-GM_50_ grout could be responsible for the observed behaviour. The lowest slum-flow value was measured for the GM_100_ composition. A high water-content reduction of 20% in the GM_100_ composition was needed to obtain the required workability of the grout; it appears that complete elimination of the limestone filler particles significantly changed the rheology of the lightweight grout. From these results, we can conclude that there is no clear relationship between the workability test and the mini-slump-flow test results. 

**Figure 3:**
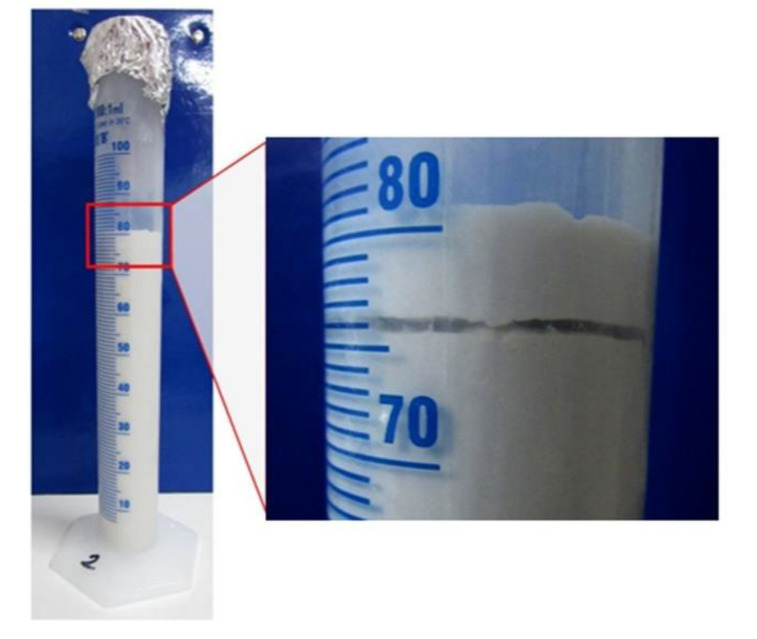
Water trapped between the two layers of the grout sample for the GM_100_ mixture.

The grouts with limestone-filler content representing 50% or more of the total filler content (LS_100_, LS_67_-GM_33_, LS_50_-GM_50_) showed a higher level of final bleeding, which ranged between 1.5% and 1.7%. In the mixtures where the prevailing part of the filler was composed of glass microspheres (LS_33_-GM_67_ and GM_100_), the final bleeding was between 0.1% and 0.6%. In all tested grouts, the final bleeding was lower than the standard limit value of 2% (EN 447 [34]; Table 1). These final bleeding values alone, however, are not enough when assessing the stability of the lightweight grout; important information can be provided by visual inspection of the sample appearance, as was the case for the GM_100_ grout (Figure 3), where it’s lowest final bleeding of 0.1% was due to the fact that a big part of the bleed water was trapped between two layers of the tested sample. The trapped water was not considered when calculating the bleeding value; such behaviour of the hydrated lime grout was observed for the first time. It appears that local internal segregation of glass microspheres, bleed water, and (possibly) lime particles happened in the test sample. Internal segregation of particles was difficult to prove due to the same white colour of the lime, limestone filler, and glass microspheres.

The results of water-retention capacity range between 78% and 84% for all tested grouts (Table 4). Although the reference grout mixture (LS_100_) seems to possess the highest water-retention capacity, the incorporation of glass microspheres did not significantly reduce this fresh grout property. Due to its high water retention, the grout resists releasing water into the highly porous media with high absorption capacity through which it flows. Consequently, the plugging of the grout inside the plasters can be prevented, and its drying shrinkage can be efficiently reduced [5]. The highest water retention was measured in the LS_100_ grout, which lacked the glass microspheres; this could be due to a lower content of free water, although this composition was prepared with the highest water content. The limestone filler particles are much finer than glass microspheres, and their shape is the same as the shape of crushed limestone aggregate grains. Thus, a significant reduction of free water content inside the LS_100_ grout can be attributed to a much higher surface at the same volume of particles (a spherical shape results in the lowest surface at a particular volume) and a higher ability of the limestone particles to capture water by adsorption and absorption. Another influencing parameter is the ability of the filler to increase the packing density of the grout’s solid particles, reducing the free water content. Ince et al. [35] showed that the filler with an appropriate granular composition could optimally fill the voids within the grout matrix. As a result, less free water would be available in the mixture during the suction action provided by porous plaster; the free water could be easily removed from the grout.

**Figure 4:**
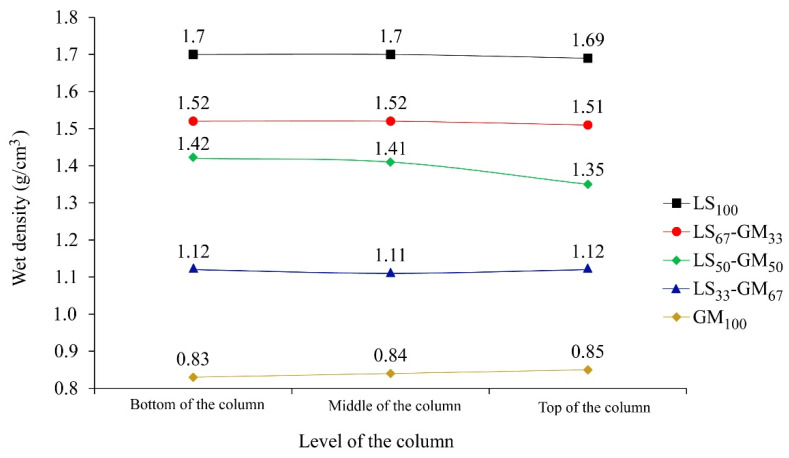
Results of the stability tests: measured grout’s wet density from the bottom, middle, and top level of the testing column.

The results of the stability test are presented in Figure 4. For the grouts LS_100_, LS_67_-GM_33_, LS_33_-GM_67_, and GM_100_ the differences in the grout’s wet density between the bottom and the top level of the testing column are low and equal to 0.01 or 0.02 g/cm^3^. All measured densities are also in agreement with density values given in Table 4. Therefore, these grouts can be evaluated as stable. When observing the grout LS_50_-GM_50_, segregation of particles was noted. The highest density was present in the bottom third of the column (1.42 g/cm^3^), while the lowest was in the top third of the column (1.35 g/cm^3^). The interparticle forces in this composition were not strong enough to maintain a homogenous suspension of particles along the column height. Therefore, a higher percentage of the limestone filler particles settled towards the bottom of the column, while a larger amount of the light glass microspheres was able to rise towards the surface. The same behaviour was also observed during the mini-slump-flow test of the LS_50_-GM_50_ grout. According to Rickerby et al. [7], the spherical morphology of glass microspheres and their coarser grain-size distribution may worsen the packing density of the composite filler. It seems that this was the case for the LS_50_-GM_50_ composition. Injection grouts have to possess sufficient stability/homogeneity after mixing, during the whole injection process, and while setting is taking place. If the mixture segregates during the process of injecting or setting, the consolidation of air pockets cannot be successful. In their study, Miltiadou-Fezans and Tassios [36] concluded that, for each grout, the critical water-to-solids ratio resulting in segregation depends on an acceptable degree of instability, the specific surface of solids, and the percentage of superplasticiser used. Based on the trapped water detected following the bleeding test (Figure 3), high stability of the fresh GM_100_ grout is an unexpected result. One possible explanation for the fresh properties measured in the GM_100_ composition is a distributed segregation of solid particles; along the entire column, there can be a local settlement of lime-binder particles, as well as flowing of the glass microspheres towards the internal-bleed water surface. 

**Figure 5:**
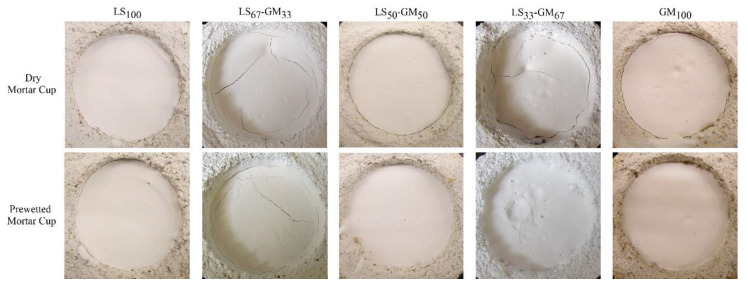
Grout mixture after drying in the dry and prewetted mortar cups.

Figure 5 and Table 5 show the results of the drying shrinkage test inside the dry or prewetted mortar cup. From the results, it is clear that the resistance of the grout to drying shrinkage and, thus, to the formation of the separation ring and cracks inside of the grout, highly depends on the filler composition used. The lowest cracking was observed for grouts LS_100_ and GM_100_, where a separation ring with a thickness of only 0.5 mm was formed in the dry cups; in the prewetted cups, the 0.5 mm separation ring was only formed in the LS_100_ composition. On the other hand, compositions with composed limestone particles and glass microspheres filler showed a weaker resistance to drying shrinkage; this was also expressed through the formation of cracks inside of the grout, observed in the LS_67_-GM_33_ (dry and prewetted mortar cup) and LS_33_-GM_67_ (dry mortar cup) compositions. It can be concluded that the combination of limestone particles with high density and modulus of elasticity and glass microspheres with extremely low density and modulus of elasticity, induces additional differential deformations in the grout that result in reduced resistance to the formation of cracks. On the other hand, a reduction of the water-to-binder ratio through the increase of the glass microspheres content decreases the sensitivity of the grout to shrinkage. These two influencing parameters with opposite effects are responsible for the observed response to drying shrinkage in each particular grout. Additionally, prewetting the mortar cups seems to be more efficient for compositions where the glass microspheres content in the filler is 50% or higher. 

The separation ring between the mortar cup and the grout and/or the cracks in the grout might indicate an excessive water content in the mixture, which could weaken the bond between the grout and the plaster layers and reduce the grout strength [17].

The grout mixtures GM_100_ and LS_50_-GM_50_ did not meet the requirements set for fresh grout properties. Due to their resistance to segregation not being high enough, we did not determine the injectability and hardened properties for these two mixtures.

**Figure 6:**
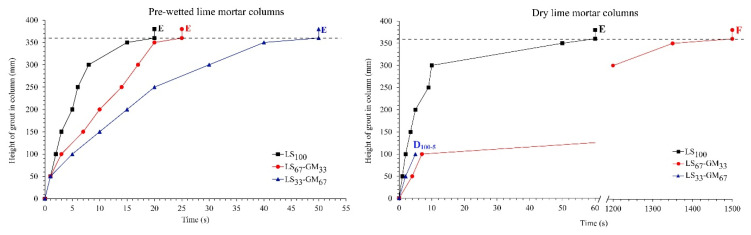
Injectability curves of the grout mixtures LS_100_, LS_67_-GM_33_, and LS_33_-GM_67_ for prewetted (left) and dry (right) lime mortar columns.

The injectability curves of the grout mixtures LS_100_, LS_67_-GM_33_, and LS_33_-GM_67_ are given in Figure 6 for the prewetted and dry crushed lime mortar columns. From these curves, it can be noted that the glass microspheres have an essential influence on the ability of the grout to be injected into detached plaster; the increase in the volume of the microspheres decreases the injectability of the grout. The results obtained are not in line with the results of studies carried out by Zajadacz and Simon [4] and Rickerby et al. [7], where glass microspheres improved the injectability of tested grouts. The authors concluded that the improvement is due to the spherical morphology and small particle size of the glass microspheres. However, in [4] there is no information regarding detailed grout composition and mixing procedure, and the composition of earthen grout used in [7] is not comparable with the hydrated lime grout used in our study. 

The results also demonstrated that prewetting of the crushed lime mortar improved the injectability of all three tested grouts. The ability of the grouts to be injected was classified following the proposal of Biçer-Şimşir and Rainer [37]. The mixtures LS_100_, LS_67_-GM_33_, and LS_33_-GM_67_ were classified as E (easy) when prewetted crushed mortar column was used. Additionally, the LS_100_ and LS_67_-GM_33_ mixtures were classified as E (easy) and F (feasible), respectively, and the LS_33_-GM_67_ mixture as D (difficult), when dry mortar column was used.

Lower bleeding and higher water-retention capacity of the LS_100_ mixture (Table 4) are the main influencing parameters responsible for better injectability of the grout not containing the glass microspheres.

### 3.2. Properties in Hardened State 

Physical properties of 90-day-old hardened grouts are given in Table 6, in the form of an average value and associated standard deviation. Measured density was the highest for the LS_100_ grout (average value of 1.45 g/cm^3^), which contained no glass microspheres, and the lowest for the LS_33_-GM_67_ grout (average value of 0.85 g/cm^3^) with microspheres occupying 2/3 of the filler volume. This shows that reducing the grout’s weight by up to (approximately) 40%, in relation to the reference LS_100_ grout, can be achieved by replacing part of the limestone filler content with the same volume of glass microspheres. When comparing the fresh and hardened state densities of a particular grout, it is obvious that the drying of the grout is responsible for the reduction in density; this reduction is equal to 0.28 g/cm^3^, 0.31 g/cm^3^, and 0.29 g/cm^3^ for the LS_100_, LS_67_-GM_33_, and LS_33_-GM_67_ grout, respectively. 

The reductions are in good correlation with the capillary porosities of the grouts (Table 6), which would be expected due to the evaporable water being held in the capillary pores. The average capillary porosities of the three grouts are in a narrow range between 38% and 40%, despite the relatively large differences in their binder-to-water ratios. Said ratios are equal to 1.86, 1.76, and 1.52 for the LS_100_, LS_67_-GM_33_, and LS_33_-GM_67_ grout, respectively. The water absorption ability of the two filler materials needs to be addressed to explain these apparent inconsistencies of properties. The water absorption of the limestone filler is equal to 0.5%, and the water content of the product is less than 0.2%. Glass microspheres are nonporous; thus, they do not absorb water. The highest part of the added water was, therefore, absorbed by the filler particles in the LS_100_ grout and the lowest by the LS_33_-GM_67_ grout particles; as a result, the narrow interval of the capillary porosities was obtained. Total porosity is the sum of the capillary pores and air pores. In the grout compositions containing glass microspheres, the spheres with broken glass walls can contribute to the measured air pores. The contribution of the glass spheres to the measured air pores’ content only appeared to be significant in the LS_33_-GM_67_ grout, which contained a high amount of glass microspheres. It seems that, during the mixing and/or the test execution, some glass microspheres may have become damaged, which is in line with the backscattered electron images in [8].

The amount of capillary water absorbed by the mixtures at the end of the test (after 24 h; W_24_) is approximately the same for the three grout compositions, resulting in the W_24_ coefficient average values between 0.42 and 0.46 kg/(m^2^√min). Obtained values are considerably lower than values given by Veiga [11] for the hydrated lime: sand (1:3) historic mortars, where W_24_ is in the range between 1.1 and 1.6 kg/(m^2^√min). However, considering the requirement that the capillary water absorption of the grout must lie between 50% and 100% of the substrate mortar W_24_ [4], the obtained results are not far from meeting the required values. Another essential property of the grout is the initial water absorption, presented by the coefficient of capillary water absorption after 10 min [1,38]. From the results in Table 6, it is evident that the average initial water absorption of the three grouts (W_10_) is approximately the same and ranging between 2.11 and 2.20 kg/(m^2^√min). These coefficients are within the W_10_ interval for the fine and coarse lime mortars prepared using Slovenian hydrated limes and limestone sands, where values range between 1.10 and 2.60 kg/(m^2^√min) [1,39]. 

The average value of the grouts’ water-vapour resistance is lower or equal to 16 (Table 6), which is in line with the results obtained for lime-based mortars by Jornet et al. [38]. The grouts LS_100_ and LS_67_-GM_33_, with the highest contents of limestone filler, showed a slightly increased water-vapour resistance (16 and 15) compared to the grout LS_33_-GM_67_ (µ = 12). Broken glass microspheres may be responsible for the obtained result.

Compressive and splitting tensile strengths are related to the total porosity; higher total porosity results in lower mechanical strength. That said, the total porosity values of the LS_67-_GM_33_ and LS_33_-GM_67_ grouts in Table 6 are underestimated due to the test method applied, which was unable to measure actual hollow volume inside of the glass microspheres. A higher actual total porosity than the one measured is evident from the densities of the grouts in the hardened state (Table 6). 

The average values for mechanical strengths are presented in Table 7, along with the corresponding standard deviation. As expected, the glass microspheres decreased the compressive and splitting tensile strengths of the grouts considerably, compared to the reference LS_100_ composition. At the ages of 90 and 365 days, the average compressive strengths of the LS_100_, LS_67_-GM_33_, and LS_33_-GM_66_ grouts were 3.5 and 3.8 MPa, 1.8 and 2.3 MPa, and 1.4 and 1.4 MPa, respectively. This means that a reduction in compressive strength between 40% and 50% can be expected when replacing a third of the limestone filler volume with glass microspheres. When the replacement is increased to two-thirds, the same reduction goes up to about 60%. While the reference grout LS_100_ complies with the proposed range of compressive strengths given by Ferragni et al. [10] for hydraulic lime grouts (Table 1), the two compositions with the glass microspheres fulfil the requirements for repair lime-based mortars given by Veiga [11], where compressive strengths in the range of 0.4–2.5 MPa are proposed. 

Moreover, Pasian et al. [8] studied grouts with reduced water content; they were prepared using slaked lime, pumice powder, quartz sand, and soda–lime–borosilicate glass microspheres. At 150 days, these grouts achieved an average compressive strength ranging from 1.15 MPa to 3.08 MPa. These values are in line with the LS_67_-GM_33_ and LS_33_-GM_66_ compressive strengths in Table 7.

The injection grouts for stabilisation of detached plaster layers are expected to fail predominantly due to tensile stresses [17]. Their tensile strength should be lower than the tensile strength of the original plaster in order to prevent the occurrence of damage to the original material [5]. The average splitting tensile strength of tested grouts at the age of 90 days was between 0.08 and 0.16 MPa (Table 7). These values are well below the 0.3–1.2 MPa range proposed by Ferragni et al. [10]. On the other hand, they fulfil the requirement given in [5] and are close to values reported by Pasian et al. [8] for the nonstructural slaked lime grout and Veiga [11] for the rendering and plastering repair mortar for historic buildings.

The main influencing parameter governing the strength properties is the volume of the grout’s solid constituents that can transfer stresses inside of the material; this is reflected in the grout’s density and porosity. There are, however, additional parameters that contribute to the strength increase. The results show that grouts with higher limestone-filler content possess higher strength, due to their lower total porosity and better interlocking between the lime binder and the filler particles. The limestone filler is a compact carbonate with sharply cornered grains and a rough surface, which can absorb up to 0.5% of the water from the fresh grout. With water, some lime particles can also be absorbed, making the bond strength between the limestone filler and lime binder considerably higher compared to that between nonabsorbent glass microspheres and lime binder. This finding is supported by the study conducted by Lanas and Alvarez [40], where they concluded that the shape of grains, particle size distribution, and chemical and mineralogical composition of the filler influence the strength of grouts. 

The mechanical strength and stiffness of the injection grout and historic lime plaster or render should be approximately equal in order to ensure adequate ductility and durability of the system. From the results in Table 7, it is evident that the glass microspheres are an efficient filler that can be used to adapt mechanical properties of the grout to the mechanical properties of historic plaster or render.

**Figure 7:**
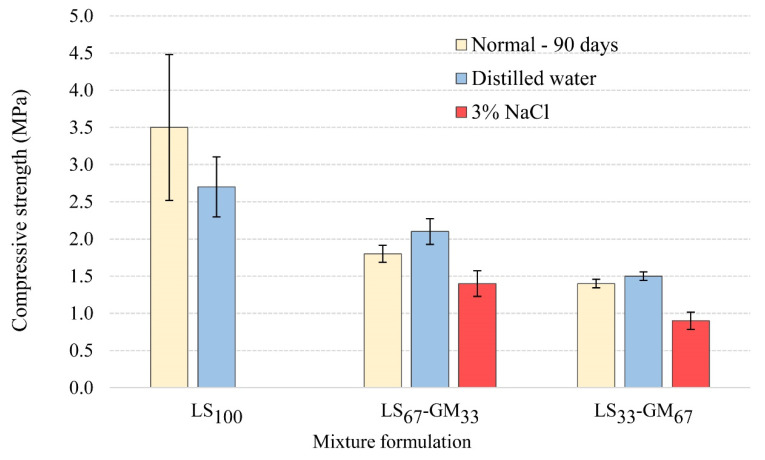
Comparison of compressive strengths of injection grouts at the age of 90 days (normal) and after the accelerated ageing using distilled water or the salt solution.

In addition to physical and mechanical properties of the hardened grouts, the durability of the grout mixtures needs to be addressed as well. Besides being an efficient weight-reducing filler, glass microspheres can be seen as a means to introduce stable micro air bubbles to the lime grout. These bubbles can increase the grout’s resistance to extreme temperature fluctuations, such as freezing and thawing during the winter and heating and cooling during the summer. The comparison of average compressive strengths for mixtures LS_100_, LS_67_-GM_33_, and LS_33_-GM_67_, at the age of 90 days and after the accelerated ageing using distilled water or de-icing salt (3% NaCl), is given in Figure 7.

The accelerated ageing of samples in the presence of distilled water shows that the glass microspheres increased the grout’s resistance to the freezing–thawing and heating–cooling cycles. While the LS_100_ grout was damaged during the accelerated ageing and, as a result, the compressive strength was decreased from the reference value of 3.5 MPa to 2.7 MPa, compositions LS_67_-GM_33_ and LS_33_-GM_67_—which contained the glass microspheres—were not damaged. Following ageing, the average compressive strength of the LS_67_-GM_33_ grout increased from the reference value of 1.8 MPa to 2.1 MPa, while that of the LS_33_-GM_67_ grout increased from 1.4 MPa to 1.5 MPa. Accelerated carbonation of the lime binder, due to wetting and drying, is most probably responsible for the observed strength increase. Similar behaviour was observed by Uranjek and Bokan-Bosiljkov [41] for lime mortar exposed to freezing and thawing cycles.

**Figure 8:**
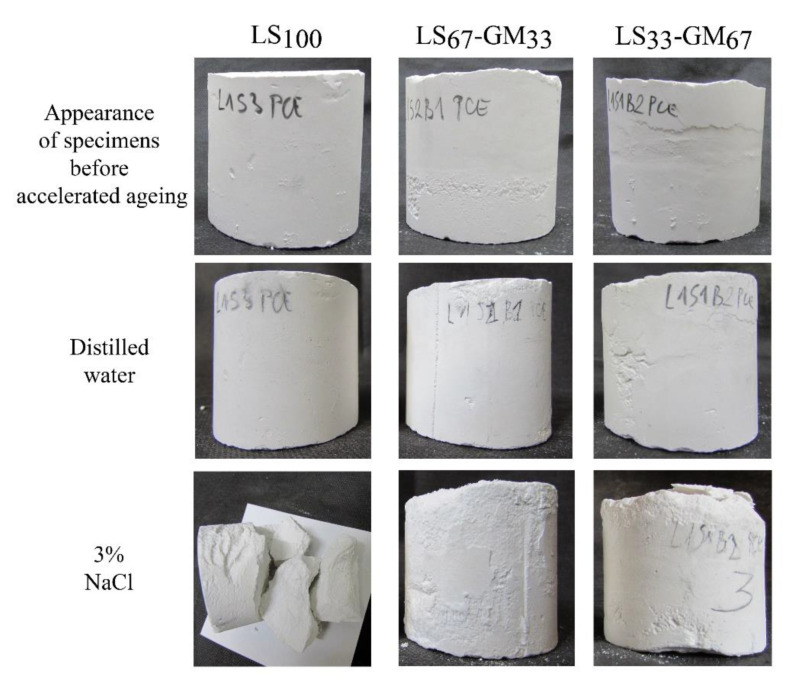
Specimens of mixtures LS_100_, LS_67_-GM_33_, and LS_33_-GM_67_, saturated in the distillate water or the de-icing agent (3% NaCl), before and after freezing–thawing and heating–cooling cycles.

When the de-icing salt solution was used for the accelerated ageing, grout LS_100_ fell apart due to the combined effect of water freezing and salt crystallisation (Figure 8). Specimens made from the LS_67_-GM_33_ and LS_33_-GM_67_ grouts, on the other hand, had retained their shape but were damaged. Dusting, swelling, scaling, and formation of cracks appeared in the lower part of the LS_67_-GM_33_ and LS_33_-GM_67_ specimens after the sixth cycle of freezing–thawing and heating–cooling (Figure 8). The compressive strength of the LS_67_-GM_33_ grout was reduced to 1.4 MPa (22% reduction), while that of the LS_33_-GM_67_ grout was reduced to 0.9 MPa (36% reduction).

From the obtained results, it is evident that the durability of tested lime grouts is much higher when their ageing takes place in the presence of pure water, compared to using de-icing salt solution; the specimens aged using distilled water did not show visible damages (Figure 8). Moreover, the compressive strengths of the LS_67_-GM_33_ and LS_33_-GM_67_ grouts improved after ageing. We can conclude that the tested grouts are highly durable solutions that can consolidate detached plasters or renders if salt-induced problems are not present. The combination of ice formation and salt crystallisation is highly detrimental to the three grouts. However, by incorporating air bubbles in the lime grout using glass microspheres, high enough durability can still be obtained for applications where salts are present in the masonry walls. The highest durability in the presence of salts was obtained for the LS_67_-GM_33_ grout, which shows that adequate balance of loadbearing capacity and micro air bubbles’ volume is needed to provide adequate durability of the lime grout in an environment containing salts. We can conclude that glass microspheres have the same function as air bubbles in aerated cement mortars; they efficiently reduce the stresses arising from water freezing inside the hardened lime grout and, thus, prevent extensive damage to the grout.

The pull-off strengths, with information about the location of failure in the panel sandwich test, are presented in Table 8. The measured pull-off strength of each particular grout is smaller than its splitting tensile strength (Table 7). The pull-off strength of the LS_100_ grout in the 2 mm air pocket achieved the value of 0.1 MPa, which is lower than the cohesive strength of lime plaster (0.15 MPa). The failure was predominantly within the grout and partly along the interface between the grout and the fine plaster. The LS_100_ grout fulfilled the pull-off strength requirement given in Table 1.

The LS_67_-GM_33_ and LS_33_-GM_67_ grouts injected in the 2 mm air pocket showed a lower average pull-off strength of 0.08 and 0.07 MPa, respectively; the requirement given in Table 1 was subsequently not met. For the two grouts, failure was predominantly along the interface between the grout and the lower rough layer of the lime plaster. This shows that the bond between the lime plaster and the grout is the weakest link in the sandwich specimen consolidated using grouts with glass microspheres.

Pull-off tests carried out on panel sandwiches with thicker air pockets (5 mm) resulted in considerably reduced pull-off strengths; in the case of the LS_100_ and LS_67_-GM_33_ grouts, they were equal to 0.05 MPa. The failure was predominantly along the interface between the base and the rough plaster. The LS_33_-GM_67_ grout sandwich specimens already failed during the test disc installation. This suggests that the drilling of the specimens may have damaged the contacts between different layers of the sandwich panels. Subsequently, measured pull-off values can highly underestimate the actual bond strength between the grout and the plaster.

When comparing results in Table 8 with the pull-off strengths reported in comparable studies [6,12], all the values measured in this study were higher. In Pasian et al. [6] the pull-off strength was in the range of 0.032–0.041 MPa at 150 days, and, in Azeiteiro et al. [12], the maximum pull-off strength after 60 and 90 days was 0.015 MPa and 0.04 MPa, respectively.

## 4. Conclusions

This study aimed to develop lightweight hydrated lime injection grouts to be used for consolidation of larger decorative plaster detachments with historical value. A reduction of the grout’s self-weight was performed by using glass microspheres as one of its constituents. In the 1:3 (volume ratio) hydrated lime: mineral filler grout, five volume ratios of limestone filler (LS) to glass microspheres (GM) were studied: 100%:0%; 67%:33%; 50%:50%; 33%:67%, and 0%:100%. Adequate workability was evaluated by a conservator—via injection of the grout through a syringe—and was set as the requirement for the grouts in the fresh state. Based on the test results obtained, the following main conclusions were drawn:

1. The glass microspheres are an efficient solution when a lower self-weight of the grout is required. However, in order to maintain the required workability and stability of fresh grout, the added water needs to be reduced when increasing the volume of glass microspheres. The effect of the glass microspheres on the rheological properties of fresh grout seems to be similar to that of air bubbles produced using air-entraining chemical admixture; they decrease yield stress and plastic viscosity of the grout.
2. The water-retention capacity of the tested grouts did not differ much between compositions, but the highest values were observed for the LS_100_ and GM_100_ grouts, which contained only one filler. This observation appears to be important when prewetting of the detached plaster is not feasible; namely, drying shrinkage of the grouts in dry mortar cups resulted in a thicker separation ring and/or crack formation in the LS_67_-GM_33_, LS_50_-GM_50_, and LS_33_-GM_67_ compositions, compared to the LS_100_ and GM_100_ grouts. On the other hand, induced differential deformations, as a result of the very different moduli of elasticity of the limestone filler and glass particles, may have also influenced the damage pattern observed.
3. The packing density of solid particles (lime, limestone filler, and glass microspheres) in the grout seems to be an essential property controlling fresh-grout stability. The LS_50_-GM_50_ and GM_100_ grouts were not stable enough to be applied in practice. On the other hand, compositions LS_67_-GM_33_ and LS_33_-GM_67_ fulfilled the stability requirement, as did the reference LS_100_ grout. As a result, further tests were performed only on these three grout compositions.
4. When the volume of glass microspheres in the grout was increased, the grout’s injectability deteriorated. The reduction of injectability was not an issue for the prewetted lime mortar column in the testing assembly; all three compositions were classified as easily injectable (E). Problems arose when the dry lime mortar column was applied; in this version of the test, only the reference LS_100_ grout was classified as E. The LS_67_-GM_33_ grout was classified as feasibly injectable (F), and LS_33_-GM_67_ as difficultly injectable (D).
5. The average dry density of the hardened grouts was reduced from 1.45 g/cm^3^ (LS_100_) to 1.20 g/cm^3^ (LS_67_-GM_33_) and 0.85 g/cm^3^ (LS_33_-GM_67_). With the reduction of the grout’s self-weight, its strength properties decreased as well. For example, the one-year average compressive strength decreased from 3.8 MPa (LS_100_) to 2.1 MPa (LS_67_-GM_33_) and 1.4 MPa (LS_33_-GM_67_). We can conclude that by replacing part of the limestone filler with the glass microspheres, an adaptation of the grout’s mechanical properties to the same properties of a particular historic plaster or render is possible. Disregarding the LS_100_ compressive strengths, which reach values typical of hydraulic lime grouts, all other measured strengths seem to fulfil requirements set for compatibility with historic lime plasters and renders.
6. The requirement of equal workability of the fresh grouts resulted in approximately the same capillary porosity and capillary water absorption properties of the LS_100_, LS_67_-GM_33_, and LS_33_-GM_67_ grouts.
7. When hydrated lime grout is exposed to extreme temperature fluctuations, such as freezing and thawing during the winter, and heating and cooling during the summer, glass microspheres inside the grout act similarly to the air bubbles (produced using an air-entraining agent) inside cement-based materials. The glass microspheres prevent entirely the damage resulting from the freezing of water inside of the grout. Moreover, they can accumulate part of the salts that crystallise within the grout and, thus, considerably reduce the damage due to salt crystallisation. It can be concluded that the glass microspheres are not only efficient in reducing the grout’s density but can also significantly improve the grout’s durability in extreme environments.

## Figures and Tables

**Table 1:** Requirements for nonstructural lime-based grout [1].

Property	Requirement
Consistency	Fluid enough to inject [4]
Bleeding	≤2% (EN 447:1996)
Shrinkage	As small as possible and <4% [10]
Wet and dry density	As low as possible
Capillary water absorption coefficient	50–100% of substrate mortar [4]
Water-vapour resistance factor (μ)	50–100% of substrate mortar [4]
Compressive strength	Lower than that of substrate (<60%) [4]
	3.0–8.0 MPa [10]
	0.4–2.5 MPa [11]
Splitting tensile strength	0.3–1.2 MPa [10]
	Lower than that of substrate [5]
Pull-off-strength	≥0.1 MPa [4]
	0.1–0.3 MPa or cohesive rupture [12]

**Table 2:** Chemical composition of the used hydrated lime (CL 90-S) and limestone filler (LS).

Compound	CL 90-S	LS
SiO_2_	2.14	<0.01
Al_2_O_3_	0.58	0.15
Fe_2_O_3_	0.20	0.01
CaO	71.01	55.38
MgO	3.05	0.76
K_2_O	0.05	0.01
Na_2_O	0.02	<0.01
SO_3_	0.14	0.01
LOI	23.38	44.02

**Table 3:** Composition of tested injection grout mixtures.

Mixture Formulation	LS_100_	LS_67_-GM_33_	LS_50_-GM_50_	LS_33_-GM_67_	GM_100_
Binder: filler (limestone filler and glass microspheres) volume ratio	1:3	1:3	1:3	1:3	1:3
Binder/filler (limestone filler and glass microspheres) mass ratio	0.28	0.40	0.54	0.85	6.19
Limestone filler: glass microspheres volume ratio	3:0	2:1	1.5:1.5	1:2	0:3
Water/binder mass ratio	1.86	1.76	1.59	1.52	1.48
Water/(binder, limestone filler and glass microspheres) mass ratio	0.41	0.50	0.56	0.70	1.28
PCE-SP (%)	0.5	0.5	0.5	0.5	0.5

Note: Sample formulation is indicated by the following symbols: LS = limestone filler; GM = glass microspheres. The volume proportion (limestone filler and glass microspheres) is indicated by subscript numbers.

**Table 4:** Fresh grout properties.

Mixture Formulation	Wet Density of Fresh Grout (g/cm^3^)	Mini Slump Flow (mm)	Bleeding (%)	Water-Retention Capacity (%)
LS_100_	1.73 ± 0.05	236 ± 5	1.5 ± 0.03	83 ± 1
LS_67_-GM_33_	1.51 ± 0.04	279 ± 6	1.7 ± 0.03	80 ± 2
LS_50_-GM_50_	1.31 ± 0.03	238 ± 16	1.7 ± 0.03	81 ± 3
LS_33_-GM_67_	1.14 ± 0.06	236 ± 10	0.6 ± 0.02	81 ± 1
GM_100_	0.82 ± 0.02	217 ± 8	0.1 ± 0.00	82 ± 2

**Table 5:** Drying shrinkage in dry and prewetted mortar cups.

Mixture Formulation	Dry Mortar Cup		Prewetted Mortar Cup	
	Separation Size (mm)	Crack Size (mm)	Separation Size (mm)	Crack Size (mm)
LS_100_	0.5	0	0.5	0
LS_67_-GM_33_	0.2	0.5	0.2	0.5
LS_50_-GM_50_	1.0	0	0.1	0
LS_33_-GM_67_	1.0	0.5	0.1	0
GM_100_	0.5	0	0	0

**Table 6:** Physical properties of the LS_100_, LS_67_-GM_33_, and LS_33_-GM_67_ grouts in the hardened state: density, total, and capillary porosity, water absorption coefficient after 24 hr (W_24_) and 10 min (W_10_), and water-vapour resistance factor (µ).

Mixture Formulation	Density in the Hardened State (g/cm^3^)	Total Porosity (%)	Capillary Porosity (%)	W_24_ (kg/(m^2^√min))	W_10_ (kg/(m^2^√min))	µ (−)
LS_100_	1.45 ± 0.01	40 ± 1.0%	38 ± 1.0%	0.46 ± 0.03	2.11 ± 0.15	16 ± 1.0
LS_67_-GM_33_	1.20 ± 0.02	42 ± 2.0%	40 ± 0.4%	0.44 ± 0.02	2.20 ± 0.10	15 ± 0.9
LS_33_-GM_67_	0.85 ± 0.00	48 ± 1.0%	38 ± 0.2%	0.42 ± 0.01	2.14 ± 0.06	12 ± 0.2

**Table 7:** Compressive strength and splitting tensile strength of the grout mixtures.

Mixture Formulation	Average Compressive Strength 90 Days (MPa)	Average Compressive Strength 365 Days (MPa)	Average Splitting Tensile Strength 90 Days (MPa)
LS_100_	3.5 ± 0.3	3.8 ± 0.4	0.16 ± 0.04
LS_67_-GM_33_	1.8 ± 0.2	2.1 ± 0.6	0.11 ± 0.07
LS_33_-GM_67_	1.4 ± 0.1	1.4 ± 0.1	0.08 ± 0.02

**Table 8:** The pull-off strengths of LS_100_, LS_67_-GM_33_, and LS_33_-GM_67_ grouts injected into the simulated air pockets with thicknesses of 2 and 5 mm.

Mixture Formulation	2 mm (MPa)	Location of Failure	5 mm (MPa)	Location of Failure
LS_100_	0.10 ± 0.02	60% within the grout 40% along the grout–fine plaster interface	0.05 ± 0.03	20% within the grout 80% along the base–rough plaster interface
LS_67_-GM_33_	0.08 ± 0.02	15% within the grout 80% along the grout–rough plaster interface 5% within the rough plaster	0.05 ± 0.01	35% within the grout 60% along the base–rough plaster interface 5% within the rough plaster
LS_33_-GM_67_	0.07 ± 0.01	95% along the grout–rough plaster interface 5% within the rough plaster	-	
Area without the grout	0.15 ± 0.02			

## References

[1] Padovnik A., Piqué F., Jornet A., Bokan-Bosiljkov V. (2016). Injection Grouts for the Re-Attachment of Architectural Surfaces with Historic Value—Measures to Improve the Properties of Hydrated Lime Grouts in Slovenia. Int. J. Archit. Herit..

[2] Pachta V., Papadopoulos F., Stefanidou M. (2019). Development and testing of grouts based on perlite by-products and lime. Constr. Build. Mater..

[3] Stefanidou M. (2014). Cement-based renders with insulating properties. Constr. Build. Mater..

[4] Zajadacz K., Simoon S. Grouting of architectural surfaces–The challenge of testing. Proceedings of the International Seminar Theory and Practice in Conservation.

[5] Biçer-Simsir B., Griffin I., Palazzo-Bertholon B., Rainer L. (2009). Lime-based injection grouts for the conservation of architectural surfaces. Rev. Conserv..

[6] Pasian C., Pique F., Jornet A., Cather S.A. (2019). Sandwich Specimen Preparation and Testing Procedure for the Evaluation of Non-Structural Injection Grouts for the Re-Adhesion of Historic Plasters. Int. J. Archit. Herit..

[7] Rickerby S., Shekede L., Zaixuan F., Wei T., Hai Q., Jinjian Y., Pique F., Agnew N. (2010). Development and Testing of the Grouting and Soluble-Salts Reduction Treatments of Cave 85 Wall Paintings. Conservation of Ancient Sites on the Silk, Proceedings of the Second International Conference on the Conservation of Grotto Sites, Mogao Grottoes, Dunhuang, China, 28 June–3 July 2004.

[8] Pasian C., Secco M., Pique F., Artioli G., Rickerby S., Cather S. (2018). Lime-based injection grouts with reduced water content: An assessment of the effects of the water-reducing agents ovalbumin and ethanol on the mineralogical evolution and properties of grouts. J. Cult. Herit..

[9] Rousset B., Gentile S., James J., Pozzi B. Injection grouts for molasse sandstones: Preliminary assessments. Proceedings of the RILEM Workshop Repair Mortars for Historic Masonry.

[10] Ferragni D., Forti M., Malliet J., Mora P., Teutonico J.-M., Torraca G. Injection grouting of mural paintings and mosaics, in Adhesives and Consolidants. Proceedings of the Preprints of the Contributions to the Paris Congress.

[11] Veiga R. Conservation of historic renders and plasters: From laboratory to site. Proceedings of the 2nd Conference on Historic Mortars-HMC 2010 and RILEM TC 203-RHM Final workshop.

[12] Azeiteiro L.C., Velosa A., Paiva H., Mantas P.Q., Ferreira V.M., Veiga R. (2014). Development of grouts for consolidation of old renders. Construct. Build. Mater..

[13] Duran A., González-Sánchez J.F., Fernández J.M., Sirera R., Navarro-Blasco Í., Alvarez J.I. (2018). Influence of two polymer-based superplasticisers (Poly-naphthalene Sulfonate, PNS, and Lignosulfonate, LS) on compressive and flexural strength, freeze-thaw, and sulphate attack resistance of lime-metakaolin grouts. Polymers (Basel).

[14] Gonzalez-Sanchez J.F., Taşcı B., Fernandez-Alvarez J.M., Navarro-Blasco I., Alvarez-Galindo J.I. (2020). Combination of polymeric superplasticisers, water repellents and pozzolanic agents to improve air lime-based grouts for historic masonry repair. Polymers (Basel).

[15] EN 459-1 (2010). Building Lime, Part 1: Definitions, Specifications and Conformity Criteria.

[16] EN 196-2 (2013). Method of Testing Cement, Part 2: Chemical Analysis of Cement.

[17] Biçer-Şimşir B., Rainer L. (2013). Evaluation of Lime-Based Hydraulic Injection Grouts for the Conservation of Architectural Surfaces: A manual of Laboratory and Field Test Methods.

[18] EN 1015-6 (1999). Methods of Test for Mortar for Masonry-Part 6: Determination of Bulk Density of Fresh Mortar.

[19] Domone P., Hsi-Wen C. (1997). Testing of binders for high performance concrete. Cement. Concrete. Res..

[20] EN 459-2 (2010). Building Lime, Part 2: Test Methods.

[21] ASTM C940-16 (2016). Standard Test Method for Expansion and Bleeding of Freshly Mixed Grouts for Preplaced-Aggregate Concrete in the Laboratory.

[22] Padovnik A., Bokan-Bosiljkov V. (2016). Adaptation of test methods used to evaluate the properties of non-structural grouts. Proceedings of the 4th Historic Mortars Conference-HMC 2016.

[23] prEN 1015-8 (1999). Methods of Test for Mortar for Masonry—Part 8: Determination of Water Retentivity of Fresh Mortar.

[24] EN 1771 (2004). Products and Systems for the Protection and Repair of Concrete Structures, Test Methods—Determination of Injectability and Splitting Test.

[25] RILEM TC 145-WSM (2002). Settlement column segregation test. The Workability and Rheology of Fresh Concrete: Compendium of Tests—Report of RILEM TC 145-WSM.

[26] EN 1015-10 (1999). Methods of Test for Mortar for Masonry, Part 10: Determination of Dry Bulk Density of Hardened Mortar.

[27] SIA 262/1 (2003). Construction En Béton-Spécifications Complémentaires, Appendix A.

[28] RILEM TC 25-PEM (1980). Test No. II. 6, Water absorption coefficient. Mater. Struct..

[29] ISO 12572 (2016). Hygrothermal Performance of Building Materials and Products—Determination of Water Vapour Transmission Properties—Cup Method.

[30] EN 1015-11 (1999). Methods of Test for Mortar for Masonry—Part 11: Determination of Flexural and Compressive Strength of Hardened Mortar.

[31] ASTM C496/C496M-1 (2004). Standard Test Method for Splitting Tensile Strength of Cylindrical Concrete Specimens.

[32] EN 1015-12 (2001). Methods of Test for Mortar for Masonry—Part 12: Determination of Adhesive Strength of Hardened Rendering and Plastering Mortars on Substrates.

[33] Bouvet A., Ghorbel E., Bennacer R. (2010). The mini-conical slump flow test: Analysis and numerical study. Cem. Concr. Res..

[34] EN 447 (2008). Grout for Prestressing Tendons. Basic Requirement.

[35] Ince C., Ozturk Y., Carter M., Wilson M. (2014). The influence of supplementary cementing materials on water retaining characteristics of hydrated lime and cement mortars in masonry construction. Mater. Struct..

[36] Miltiadou-Fezans A., Tassios T.P. (2013). Stability of hydraulic grouts for masonry strengthening. Mater. Struct..

[37] Biçer-Şimşir B., Rainer L. Field test methods for comparative evaluation of lime-based hydraulic injection grouts for the conservation of architectural surfaces. Proceedings of the ICOM-CC 17th Triennial Conference.

[38] Jornet A., Mosca C., Cavallo G., Corredig G. Comparison Between Traditional Lime Based and Industrial Dry Mortars. Proceedings of the 2nd Conference on Historic Mortars-HMC 2010 and RILEM TC 203-RHM Final Workshop.

[39] Padovnik A. (2016). Consolidation of Detached Plaster Layers of Mural Paintings with Non-Structural Grouting. Ph.D. Thesis.

[40] Lanas J., Alvarez-Galindo J.I. (2003). Masonry repair lime-based mortars: Factors affecting the mechanical behavior. Cem. Concr. Res..

[41] Uranjek M., Bokan-Bosiljkov V. (2015). Influence of freeze–thaw cycles on mechanical properties of historical brick masonry. Constr. Build. Mater..

